# Nut consumption and the risk of oesophageal squamous cell carcinoma in the Golestan Cohort Study

**DOI:** 10.1038/s41416-018-0148-0

**Published:** 2018-06-28

**Authors:** Maryam Hashemian, Gwen Murphy, Arash Etemadi, Hossein Poustchi, Maryam Sharafkhah, Farin Kamangar, Akram Pourshams, Akbar Fazeltabar Malekshah, Masoud Khoshnia, Abdolsamad Gharavi, Azita Hekmatdoost, Paul J. Brennan, Paolo Boffetta, Sanford M. Dawsey, Christian C. Abnet, Reza Malekzadeh

**Affiliations:** 1 Digestive Oncology Research Center, Digestive Diseases Research Institute, Tehran University of Medical Sciences, Tehran, Iran; 2 Metabolic Epidemiology Branch, Division of Cancer Epidemiology and Genetics, National Cancer Institute, Bethesda, MD USA; 3 Department of Nutrition and Biochemistry, Faculty of Medicine, Sabzevar University of Medical Sciences, Sabzevar, Iran; 4 Liver and Pancreatobiliary Diseases Research Center, Digestive Diseases Research Institute, Tehran University of Medical Sciences, Tehran, Iran; 5 School of Computer, Mathematical, and Natural Sciences, Morgan State University, Morgan State University, Baltimore, MD USA; 6 Digestive Disease Research Center, Digestive Diseases Research Institute, Tehran University of Medical Sciences, Tehran, Iran; 7 Golestan Research Center of Gastroenterology and Hepatology, Golestan University of Medical Sciences, Gorgan, Iran; 8 Department of Clinical Nutrition and Dietetics, Faculty of Nutrition and Food Technology, National Nutrition and Food Technology Research Institute, Shahid Beheshti University of Medical Sciences, Tehran, Iran; 9 International Agency for Research on Cancer, Lyon, France; 10 Tisch Cancer Institute, Icahn School of Medicine at Mount Sinai, New York, NY USA

**Keywords:** Risk factors, Oesophageal cancer

## Abstract

**Background:**

Nut consumption has been inversely associated with gastric cancer incidence in US-based studies, but not with oesophageal cancer. However, there is aetiologic heterogeneity, among oesophageal squamous cell carcinoma (ESCC) cases in low-risk vs. high-risk populations. The objective of this study was to evaluate the association between nut consumption and risk of ESCC in a high-risk population.

**Methods:**

The Golestan Cohort Study enroled 50,045 participants in Northeastern Iran, between 2004 and 2008. Intake of peanuts, walnuts and mixed nuts (including seeds) were assessed using a validated food frequency questionnaire at baseline. Cox proportional hazard models were used to estimate hazard ratios (HR) and 95% confidence intervals for subsequent ESCC adjusted for potential confounders. Non-consumers of nuts were used as the reference category and the consumers were categorised into tertiles.

**Results:**

We accrued 280 incident ESCC cases during 337,983 person-years of follow up. Individuals in the highest tertiles of total nut consumption, and mixed nut consumption were significantly associated with lower risk of developing ESCC compared to non-consumers (HR = 0.60, 95% CI = 0.39–0.93, p-trend = 0.02, and HR = 0.52, 95% CI = 0.32–0.84, p trend = 0.002, respectively).

**Conclusions:**

We found a statistically significant inverse association between total nut consumption and the risk of ESCC in this high-risk population.

## Introduction

Nuts appear to have beneficial effects on all cause and cancer-specific mortality,^1, 2^ and are part of the recommended Mediterranean diet.^3^ A source of polyphenols, vitamins, minerals and fibres,^4^ nuts can also be rich in monounsaturated fatty acids (MUFA) or polyunsaturated fatty acids, depending on the type of nut.^4^ Nuts are hypothesised to protect against cancer development by inhibition of cell proliferation, enzyme regulation, inhibition of oncogenes, induction of tumour suppressor gene expression, and induction of cell differentiation and apoptosis.^5^ Previous studies have demonstrated that nut consumption is associated with a decreased risk of gastric, colorectal, and pancreatic cancers,^6, 7^ and cancer-specific mortality.^1^ Although all nuts are nutritious foods, there are some differences in their vitamin, mineral and fatty acid composition. However, the effect of each type of nut has not been investigated before.

Oesophageal cancer is the sixth most common cause of cancer death worldwide.^8^ The tumour has two different subtypes: oesophageal adenocarcinoma and oesophageal squamous cell carcinoma (ESCC).^8^ ESCC is the most common type of oesophageal cancer in the world, representing more than 80% of all cases.^9^ In a recent study, nut consumption was not associated with the risk of ESCC in a cohort of middle-aged US adults.^10^ However, there is substantial aetiologic heterogeneity in ESCC between low-incidence populations, where smoking and alcohol are the predominant risk factors, and high-incidence populations, where these two agents play a much smaller role.^11^ Dietary factors are also likely to be different in low and high-incidence populations.^12^

Northeastern Iran has high rates of ESCC,^13^ and was first investigated by the International Agency for Research on Cancer and local investigators in the 1960s and 1970s.^14, 15^ In the early 2000s an international collaborative group restarted an oesophageal cancer research programme in the region that is now known as Golestan Province.^16^ An inverse association has been reported between nut consumption and total cancer mortality in the Golestan Cohort Study.^17^ The aim of the current study was to evaluate the associations between nut consumption and the risk of ESCC in this same cohort. This study provides data on different types of nuts, including peanuts, walnuts, and mixed nuts (including seeds), which allows us to evaluate the effect of each type of nut on ESCC.

## Methods

### Study population

A detailed description of the study design and methods has been published previously.^16^ In brief, 50,045 adults, aged 40 years old and above, from Golestan Province in northeastern Iran were recruited between January 2004 and June 2008. The total cohort was 42% male, 80% rural, 74% Turkman, and 70% with no formal education. At baseline, each participant completed a general questionnaire consisting of demographic and risk factor questions and a food frequency questionnaire (FFQ). After enrolment, participants were followed for vital status, major causes of death, incident cancer and cardiovascular disease. For the current analysis, the exclusion criteria were as follow: participants with missing information on nut consumption (*n* = 872), persons with extreme energy intake, defined as more than two interquartile ranges above the 75th percentile or below the 25th percentile of intake (*n* = 642), and participants who reported history of any cancer except non-melanoma skin cancer (*n* = 154) or had reported renal failure at baseline because they should not consume nuts (*n* = 93). This analysis includes the remaining 48,284 participants. All participants provided written informed consent at baseline.

The study was approved by the Institutional Review Boards of the Digestive Disease Research Institute of Tehran University of Medical Sciences, Iran; the National Cancer Institute (NCI) in the United States; and the International Agency for Research on Cancer (IARC) in France.

### Dietary Assessment

Dietary information was collected using a 116-item semi-quantitative FFQ specifically designed for this population, which has been validated against twelve 24-hour dietary recall questionnaires.^18^ Participants were asked about their consumption of common nuts in this population including “peanuts”, “walnuts” and “mixed nuts and seeds” (which in this area are typically composed of watermelon seeds, pumpkin seeds, pistachios, almonds, and other tree nuts). Hidden source of nuts and seeds are not common in this population. Subjects reported their frequency of consumption of a given serving size of each food item daily, weekly or monthly during the previous year. Daily intake of each food item was calculated by multiplying the frequency of consumption by the typical portion size and the number of servings per day. For this study, daily intake of each food item was converted to grams. We constructed a new variable “total nut consumption”, which summed the consumption of peanuts, walnuts, and mixed nuts and seeds.

### Ascertainment of end points

In our analyses, the primary endpoint was first incident ESCC. At the time of enrolment, all participants were instructed to contact the cohort team in case of any certain conditions like a new major disease or hospitalisation. In addition, participants were contacted by telephone and case review questionnaires were completed once every year. Any hospital admissions or occurrence of disease that had taken place since the previous follow-up contacts were recorded. Reports of cancers, upper GI endoscopy or death were followed by a home or hospital visits to collect all clinical reports, pathology reports and tumour samples (if available). A verbal autopsy was performed for deceased participants. Two internists reviewed all available reports and allocate a disease code for each outcome. In case of any discrepancy, a third expert internist reviewed the documents and made the final decision on the code. Since oesophageal cancer was the most important outcome of the study, all medical or pathology reports of oesophageal cancer were reviewed and verified by an Endpoint Review Committee composed of experts from the Digestive Diseases Research Institute of Tehran University. 239 out of 280 cases were histologically confirmed. Ascertainment of mortality and cancer endpoints has been described in detail elsewhere ^16^. During the period of analysis, 369 participants (0.7%) were lost to follow-up.

### Statistical analysis

Nut consumption was reported in four categories. Since 28% of participants reported no nut consumption in the FFQ during past 12 months, we made non-consumers the reference group for our analyses, and the consumers were categorised into tertiles using the grams of nuts they consumed per 1000 kcal. The median of each category (tertile) was used to assess linear trends. Nut consumption was also evaluated as a continuous variable scaled to 5 grams/day increments (approximately equal to ¼ cup increments per week). We also investigated ever vs. never nut consumers. In an additional analysis, we categorised nut consumers by quartiles, using the lowest category of consumers as the reference.

Differences in potential risk factors of ESCC in this region were examined across categories of nut consumption. A Cox proportional hazard model was used to estimate hazard ratios (HRs) and 95% confidence intervals (95% CIs). The proportional hazards assumption was tested (and upheld) using Aalen plots and the Schoenfeld residuals test. For this analysis, person-years were calculated as the time from the completion of the baseline FFQ to one of the following events: (1) diagnosis of gastrooesophageal cancer, (2) death, (3) loss to follow-up or (4) the end of follow-up for this analysis (1 November 2016), whichever came first.

Multivariable models were adjusted for known or suspected risk factors for ESCC in this population, including age (years), sex (M/F), BMI ( < 18.5, ≥18.5, ≥25, ≥30 kg/m^2^), formal education (none, any), ethnicity (non-Turkmen, Turkmen), place of residence (urban, rural), smoking status (pack-year), opium use (nokhod-year, a local unit for opium consumption that weighs about 200 mg), alcohol drinking (never, ever), physical activity at work (irregular non-intense, regular non-intense, irregular or regular intense),^19^ wealth score (a composite score including household ownership, house size, appliances, vehicles and other variables associated with wealth,^20^ categorised to tertiles), intake of fruits and vegetables (grams per 1000 Kcal) and total energy intake. Energy adjustment was conducted using an energy density model based on grams per 1000 Kcal intake, in addition to including energy in the model.^21^ When modelling risks for walnuts and peanuts, we obtained estimates for both from a single model and a joint model (mutual adjustment) to assess the independence of the effects.

We evaluated potential effect modification by age, sex, ethnicity, BMI, smoking status, opium status and alcohol drinking by including a cross-product in the model using likelihood ratio tests. For evaluating interactions among continuous variables, we used medians to categorise them. A sensitivity analysis was performed by excluding the first two years of follow-up to assess whether reverse casualty could be a concern. Other sensitivity models included: excluding participants with BMI less than 18.5 kg/m^2^ or more than 35 kg/m^2^; excluding participants in the first or last deciles of wealth score; and excluding participants who were tobacco smokers, opium users and/or alcohol drinkers. Statistical analyses were performed using STATA software (version 13, STATA Corp, College Station, TX, USA). Reported *p*-values are two-sided, and *p*-values < 0.05 were considered statistically significant.

## Results

72% of the participants reported consuming nuts. The median (IQR) intake of total nuts, mixed nuts and seeds, peanuts and walnuts were 1.89 (0.69–4.46), 1.33 (0.74–3.53), 0.83 (0.34–1.79) and 0.22 (0.09–0.71) g/day among nut consumers, respectively. Table 1 shows the baseline characteristics of participants by categories of total nut intake per 1000 kcal. Nut consumers were more likely to be younger compared to those who reported never consuming nuts. Further, they were more likely to have a higher wealth score, live in urban areas, and consume more fruits and vegetables, and they were more likely to be male, Turkman, have a higher BMI, and have formal education. They were also more likely to drink alcohol and less likely to smoke tobacco or opium. Mix nut and seed consumer, peanut consumer and walnut consumer had the same pattern for these confounders (Supplementary Table 1).

**Table 1:** Baseline characteristics of subjects by categories of total nut intake in the Golestan Cohort Study^a^

		Nut intake^b^ categories (C)			
	Total population (*N*=48,284)	No nut consumption (*N*=13,333)	Tertile 1 (*N*=11,554)	Tertile 2 (*N*=11,540)	Tertile 3 (*N*=11,857)
Median intake (g per 1000 kcal/day)	0.44	0	0.2	0.9	2.8
Age, years, mean ± SD	52.1 ± 8.9	55.8 ± 9.4	52.2 ± 8.7	50.4 ± 8.1	49.3 ± 7.7
BMI, kg/m^2^, mean ± SD	26.7 ± 5.4	25.5 ± 5.5	26.6 ± 5.4	27.1 ± 5.4	27.5 ± 5.3
Fruit intake, grams per 1000 kcal, mean ± SD	68.6 ± 51.2	52.1 ± 43.3	63.1 ± 48.6	72.6 ± 49.4	88.5 ± 55.8
Vegetable intake, grams per 1000 kcal, mean ± SD	87.2 ± 38.3	83.5 ± 40.1	85.6 ± 36.3	87.5 ± 36.0	92.6 ± 39.5
Sex, male (%)	42.3	39.9	40.7	42.9	45.2
Place of residence, rural (%)	79.7	89.6	77.3	77.1	73.5
Ethnicity, Turkmen (%)	74	68.3	72.7	76.6	79.0
Education, No formal (%)	70.1	84.8	73.1	64.4	56.4
Physical activity at work					
Irregular, non-intense (%)	62.1	72.0	61.9	58.7	54.5
Regular, non-intense (%)	26.3	16.1	25.4	29.7	35.1
Regular or irregular, intense (%)	11.6	11.8	12.6	11.6	10.4
Wealth score					
Low (%)	32.1	43.4	33.1	27.4	22.8
Medium (%)	33.6	38.0	35.1	32.4	28.5
High (%)	34.3	18.7	31.8	40.2	48.6
Ever alcohol drinker (%)	3.5	2.2	3.1	3.5	5.0
Smoking, pack-year	2.9 ± 10.1	3.5 ± 11.7	2.9 ± 10.2	2.7 ± 9.0	2.6 ± 8.7
Opium use, nokhod-year^c^	9.6 ± 49.7	13.1 ± 62.6	8.7 ± 42.9	8.1 ± 43.8	8.0 ± 44.5

^a^All covariates were associated with nut consumption with *p* < 0.001.

^b^Intake density (grams per 1000 kcal), including tree nut, peanut, walnut and seed.

^c^A local unit for opium consumption that weighs about 200 mg

A total of 280 participants developed ESCC during the study period, with a median follow up of 9 years. Table 2 shows the associations between nut consumption and risk of incident ESCC. The highest tertile of total nut consumption was associated with lower risk of developing ESCC compared to non-consumers (adjusted HR C3_vs_C0 = 0.60, 95% CI = 0.39–0.93, *p*-trend = 0.02). A similar inverse association was observed between mixed nut and seed consumption and risk of ESCC (adjusted HR C3_vs_C0 = 0.52, 95% CI = 0.32–0.84, *p* trend = 0.002). By contrast, neither peanut or walnut consumption was significantly associated with risk of ESCC (adjusted HR C3_vs_C0 = 0.80, 95% CI = 0.51–1.24 and adjusted HR C3_vs_C0 = 0.71, 95% CI = 0.45–1.14, respectively (Table 2).

**Table 2:** Crude and adjusted Hazard ratios of incident oesophageal squamous cell carcinoma, by nut intake^a^ (*N***=**48,284)

	Categories of intake^b^					Continuous HR per 5 g/1000 kcal/day increased intake
	No nut consumption	Tertile 1	Tertile 2	Tertile 3	*p*-trend^c^	
Total nuts (including tree nut, peanut, walnut and seed)						
Mean intake (g/ 1000 kcal) ± SD	−	0.21 ± 0.13	0.92 ± 0.29	3.84 ± 3.23		
Person years	114,663	106,297	107,322	111,456		
Cases, *n*	118	73	57	32		
HR, (95% CI)^d^	1	0.65 (0.48–0.87)	0.52 (0.38–0.71)	0.27 (0.18–0.40)	<0.001	0.45 (0.34–0.60)
HR, (95% CI)^e^	1	0.88 (0.66–1.19)	0.84 (0.61, 1.17)	0.49 (0.32–0.73)	0.001	0.64 (0.50–0.81)
HR, (95% CI)^f^	1	1.02 (0.75–1.39)	1.03 (0.73, 1.44)	0.60 (0.39–0.93)	0.02	0.71 (0.55–0.91)
Mixed nuts and seeds						
Mean intake (g/ 1000 kcal) ± SD	−	0.16 ± 0.09	0.61 ± 0.19	2.90 ± 2.73		
Person years	193230	82384	81460	84487		
Cases, *n*	184	49	26	21		
HR, (95% CI)^d^	1	0.63 (0.46–0.86)	0.33 (0.22–0.50)	0.26 (0.17–0.42)	<0.001	0.31 (0.19–0.49)
HR, (95% CI)^e^	1	0.91 (0.66–1.25)	0.53 (0.34–0.81)	0.47 (0.29–0.74)	<0.001	0.54 (0.36–0.80)
HR, (95% CI)^f^	1	1.21 (0.87–1.68)	0.58 (0.37–0.91)	0.52 (0.32–0.84)	0.002	0.57 (0.38–0.86)
Peanuts						
Mean intake (g/ 1000 kcal) ± SD	–	0.12 ± 0.05	0.41 ± 0.14	1.94 ± 2.10		
Person years	221830	70670	73386	74385		
Cases, *n*	177	36	41	26		
HR, (95% CI)^d^	1	0.63 (0.44–0.91)	0.71 (50–0.99)	0.43 (0.28–0.65)	<0.001	0.48 (0.30–0.78)
HR, (95% CI)^e^	1	0.83 (0.58–1.20)	1.03 (0.73–1.46)	0.66 (0.43–1.01)	0.001	0.73 (0.48–1.09)
HR, (95% CI)^f^	1	0.97 (0.67–1.41)	1.21 (0.84–1.72)	0.80 (0.51–1.24)	0.02	0.85 (0.59–1.24)
Walnuts						
Mean intake (g/ 1000 kcal) ± SD	–	0.03 ± 0.01	0.10 ± 0.04	0.86 ± 1.20		
Person years	249,012	58,468	67,979	65,866		
Cases, *n*	199	30	30	21		
HR, (95% CI)^d^	1	0.62 (0.42–0.92)	0.55 (0.38–0.81)	0.40 (0.26–0.63)	<0.001	0.07 (0.01–0.33)
HR, (95% CI)^5^	1	0.77 (0.52–1.15)	0.69 (0.47–1.01)	0.46 (0.29–0.72)	0.001	0.11 (0.02–0.46)
HR, (95% CI)^f^	1	0.89 (0.60–1.32)	0.81 (0.54–1.21)	0.71 (0.45–1.14)	0.16	0.31 (0.08–1.17)

^a^Intake density (g per 1000 kcal).

^b^The categories of intake were defined separately for each nut category.

^c^The test for trend used ordinal models with tertile mid-points as values.

^d^Crude models.

^e^Adjusted for age (years) and sex.

^f^Adjusted for age (years), sex, place of residence (urban, rural), smoking (pack-year), opium user (nokhod-year, a local unit for opium consumption that weighs about 200 mg), wealth score (low, medium, high), ethnicity (non-Turkmen, Turkmen), body mass index (<18.5, ≥18.5, ≥25, ≥30 kg/m^2^), education (no formal education, formal education), physical activity (irregular non-intense, regular non-intense, regular or irregular intense), fruits intake (g/1000 kcal) and vegetables intake (g/1000 kcal); HRs (95% CI) were calculated using Cox regression models

The ever vs. never (total nut) consumers had a decreased risk of developing ESCC (adjusted HR = 0.62, 95% CI = 0.42–0.92). However, for the individual categories of mixed nut and seed, peanut or walnut ever vs. never consumption, the inverse associations were not significant (adjusted HR = 0.80, 95% CI = 0.61–1.05, adjusted HR = 0.99, 95% CI = 0.77–1.30, adjusted HR = 0.80, 95% CI = 0.61–1.05, respectively).

Modelling nut consumption as a continuous variable, we observed a statistically significant 29% decrease in ESCC risk for each 5 g of total nuts consumed per day (Table 2). An inverse association between 5 g/day increases in peanut or walnut consumption and risk of ESCC did not reach statistical significance (Table 2). Limiting the analysis to nut consumers, the highest quartile of total nut consumption was associated with lower risk of developing ESCC compared to the lowest quartile (adjusted HR Q4_vs_Q1 = 0.59, 95% CI = 0.35–0.99).

Mutual adjustment for peanuts or walnuts did not substantially alter risk estimates (data not shown). We found no evidence of effect modification by age, sex, ethnicity, BMI, smoking status, opium status and alcohol drinking (*p* > 0.05 for all analyses). Table 3 shows the results of sensitivity analyses by excluding the first two years of follow-up; those with extreme BMI; those with extreme wealth scores; or those who were smokers, opium users and/or alcohol drinkers. None of these models suggested meaningful differences in the overall results.

**Table 3:** Sensitivity analyses of the association of increasing 5 g per day of total nut intake^a^ and risk of ESCC

Exclusion	HR^b^ (95% CI)	*P* for trend
No exclusions	0.71 (0.56–0.91)	0.03
First 2 years of follow up	0.71 (0.56–0.92)	0.04
Participants with extreme BMI^**c**^	0.74 (0.58–0.94)	0.04
Participants with extreme wealth score^d^	0.75 (0.59–0.97)	0.06
Smokers, opium users and/or alcohol drinkers	0.69 (0.51–0.95)	0.04

^a^Including tree nut, peanut, walnut and seed.

^b^Adjusted for age (years), sex (M, F), place of residence (urban, rural), smoking (pack-year), opium user (nokhod-year, a local unit for opium consumption that weighs about 200 mg), wealth score (low, medium, high), ethnicity (non-Turkmen, Turkmen), body mass index ( < 18.5, ≥18.5, ≥25, ≥ 30), education (no formal, formal education), physical activity (Irregular non-intense, Regular non-intense, regular or irregular intense), fruits intake (g/d) and vegetables intake (g/d); HRs (95% CI) were calculated using Cox regression models.

^c^BMI <18.5 or BMI >35

^d^The first and last deciles of wealth score

## Discussion

In a population with a high ESCC incidence, we found an inverse association between total nut consumption and the risk of ESCC. Similarly, we found an inverse association with mixed nuts and seeds consumption. However, the risk of ESCC was not associated with reported walnut or peanut consumption. To our knowledge, this is the first prospective study assessing the association between consumption of different types of nut and the risk of ESCC.

An inverse association between total nut consumption and cancer mortality was previously noted in a meta-analysis.^1^ In a previous study in this population in Iran, nut consumption was associated with decreased risk of total mortality and cancer-related mortality.^17^ Further, our current results are consistent with the findings in other types of cancers including colon cancer, lung cancer and pancreatic cancer.^7, 22^ By contrast, a recent study in a US cohort showed no association between nut and seed consumption with ESCC risk.^10^ Such a discrepancy between the significance of risk factors in low-risk and high-risk populations has been reported before for smoking, alcohol and sex.^11^ This aetiologic heterogeneity of ESCC is remarkable, but the reason is not clear yet.^11^

We observed a statistically significant inverse association between mixed nut and seed consumption and the risk of ESCC; we also found point estimates <1 but no significant association between peanut or walnut consumption and risk of ESCC. In Golestan, where we undertook our study, mixed nuts and seeds would usually include watermelon seeds, pumpkin seeds, pistachios and almonds. The lack of a significant effect for peanut or walnut consumption could be due to lower intake of peanuts and walnuts relative to mixed nuts and seeds in this population. However, the difference could also be explained by the different compositions of different types of nuts. Although all nuts are nutritious foods, there are some differences in their vitamin, mineral and fatty acid composition (Table 4). Among nuts and seeds, watermelon and pumpkin seeds are the best source of zinc (containing about three times more than in peanuts or walnuts).^23^ Zinc intake is inversely associated with the risk of ESCC.^19, 24, 25^ Zinc is necessary for immune function, and for transcription factors which control cell proliferation and signalling pathways.^26, 27^ Almonds are the best source of calcium and riboflavin among nuts (containing three times more calcium and eight times more riboflavin than peanuts or walnuts).^23^ Both calcium intake and riboflavin status have previously been shown to be inversely associated with ESCC risk.^19, 28^ Previous studies showed that calcium suppresses the cell cycle, and promotes apoptosis, ^29, 30^ and riboflavin deficiency suppresses immune functions and may fail to control chronic inflammation.^31^ Almonds are also a good source of MUFAs, which have anti-inflammatory effects.^23^ Peanuts are source of folate, and folate has inverse association with risk of ESCC.^32^ Folate deficiency may decrease the level of S-adenosylmethionine and consequently cause DNA hypomethylation and cancer.^32^ Eating a variety of nuts and seeds could provide an excellent source of all of these nutrients in comparison with not eating nuts or eating only one specific type of nut, and greater weight should be placed upon the consumption of watermelon and pumpkin seeds in Iran.

**Table 4:** Nutrient values of different types of nuts and seeds^a^

Nutrients	Peanuts	Walnuts	Almonds	Pumpkin seeds	Watermelon seeds	Pistachio
Energy (kcal)	567	654	579	559	557	560
Calcium (mg)	92	98	269	46	54	105
Iron (mg)	5	3	4	9	7.3	4
Magnesium (mg)	168	158	270	592	515	121
Zinc (mg)	3	3	3	8	10	2.2
Thiamin (mg)	0.6	0.3	0.2	0.3	0.2	0.9
Riboflavin (mg)	0.14	0.15	1.14	0.15	0.15	0.16
Niacin (mg)	12.1	1.1	3.6	5.0	3.5	1.3
Vitamin B6 (mg)	0.3	0.5	0.1	0.1	0.1	1.7
Folate (μg)	240	98	44	58	58	51
Saturated fat (g)	6	6	4	9	10	6
Mono-unsaturated fat (g)	24	9	31	16	7	23
Poly-unsaturated fat (g)	15	47	12	21	28	14

^a^According to USDA release 28

This study has several strengths. The Golestan Cohort is a prospective study, in a high-risk population for oesophageal cancer, with an excellent follow-up rate. Because nut consumption is high in this population, it was assessed using three separate questions (about peanuts, walnuts, and mixed nuts and seeds), in contrast to other studies, many of which used a single question to assess the consumption of nuts.^7^

Our study also has several limitations. One limitation is that some degree of measurement error is inevitable when analysing self-reported intake of foods recorded by an FFQ. Nut consumption was significantly associated with higher socioeconomic status, meaning that nut consumers may have had healthier diets because of their socioeconomic status, and this may partially explain their decreased risk of oesophageal cancer. Adjusting our analysis for wealth score, education, intake of fruits and vegetables and other known risk factors did significantly reduce the strength of all associations. Considering the difference between the crude and adjusted HRs, we cannot exclude the possibility of residual confounding. We were not able to investigate the influence of preparation method or salted vs. unsalted nuts. We also cannot conclude a causal relationship from this observational study.

In conclusion, we found an inverse association between total nut consumption and the risk of ESCC in this large Iranian cohort. This association needs to be confirmed in future studies, along with additional research on the association between nut consumption and risk of total mortality, specific causes of mortality, and incidence of other cancers to inform public health recommendations. We also suggest comparing the effect of nuts and seeds in future studies where the data are available.

## Electronic supplementary material

Supplemental material
