# Supplementary material for: Nut consumption and the risk of oesophageal squamous cell carcinoma in the Golestan Cohort Study
Source: Br J Cancer. 2018 Jun 28;119(2):176–81. doi: 10.1038/s41416-018-0148-0 (PMC6048068; doi:10.1038/s41416-018-0148-0)
Supplement: Supplementary file 1 — Supplemental material [file 41416_2018_148_MOESM1_ESM.docx]

| Supplemental Table 1. Baseline characteristics of subjects by categories of various nut intake^1^ in the Golestan Cohort Study^2^ | | | | | | | | | | | | |
| --- | --- | --- | --- | --- | --- | --- | --- | --- | --- | --- | --- | --- |
|  | **Mixed nuts and seeds intake categories** | | | | **Peanut intake categories** | | | | **Walnut intake categories** | | | |
|  | **No nut consumption** | **Tertile 1** | **Tertile 2** | **Tertile 3** | **No nut consumption** | **Tertile 1** | **Tertile 2** | **Tertile 3** | **No nut consumption** | **Tertile 1** | **Tertile 2** | **Tertile 3** |
| Median intake ^3^ | 0 | 0.15 | 0.58 | 1.96 | 0 | 0.12 | 0.39 | 1.29 | 0 | 0.03 | 0.09 | 0.51 |
| Age, years, mean±SD | 54.9±9.4 | 50.7±8.1 | 49.5±7.7 | 48.7±7.2 | 53.8±9.3 | 60.0±8.3 | 50.0±38.1 | 49.4±7.8 | 52.9±9.2 | 50.8±8.2 | 50.6±8.4 | 51.3±8.6 |
| BMI, kg/m^2^, mean±SD | 25.9±5.5 | 27.3±5.4 | 27.2±5.4 | 27.4±5.3 | 26.1±5.5 | 27.0±5.4 | 27.2±5.3 | 27.5±5.4 | 26.2±5.5 | 27.0±5.4 | 27.1±5.3 | 27.7±5.2 |
| Fruit intake ^3^, mean±SD | 59.5±46.7 | 70.4±52.8 | 74.0±51.9 | 83.7±54.6 | 58.8±46.7 | 68.6±50.8 | 79.3±53.6 | 89.1±54.4 | 58.4±46.6 | 68.6±46.7 | 79.0±48.3 | 97.7±60.7 |
| Vegetable intake ^3^, mean±SD | 85.8±39.9 | 86.9±37.7 | 87.8±35.1 | 90.3±37.4 | 85.6±39.0 | 86.3±36.1 | 88.3±36.6 | 91.9±39.1 | 83.9±37.3 | 84.6±34.0 | 90.1±36.5 | 99.5±44.1 |
| Sex, male (%) | 40.5 | 41.7 | 43.6 | 45.1 | 40.3 | 42.1 | 45.7 | 44.4 | 41.3 | 42.3 | 43.3 | 43.8 |
| Place of residence, rural (%) | 86.4 | 67.5 | 76.7 | 78.3 | 86.1 | 71.4 | 74.2 | 72.7 | 87.0 | 76.6 | 74.5 | 59.4 |
| Ethnicity, Turkmen (%) | 71.3 | 69.5 | 76.8 | 82.2 | 71.1 | 73.6 | 79.0 | 78.6 | 74.4 | 77.8 | 74.9 | 68.2 |
| Education, No formal (%) | 80.4 | 63.7 | 61.1 | 60.1 | 79.0 | 66.4 | 60.9 | 54.8 | 77.8 | 68.1 | 61.2 | 51.5 |
| Physical activity at work, intense | 11.4 | 11.7 | 12.3 | 11.4 | 12.5 | 11.1 | 11.2 | 9.7 | 13.1 | 11.4 | 10.0 | 7.7 |
| Wealth score, high | 25.1 | 39.5 | 42.0 | 44.3 | 25.8 | 37.3 | 42.9 | 50.2 | 24.2 | 36.7 | 46.6 | 59.2 |
| Ever alcohol drinker (%) | 2.7 | 3.9 | 3.7 | 4.5 | 2.5 | 3.9 | 4.1 | 5.2 | 2.5 | 3.7 | 4.4 | 5.9 |
| Smoking, pack-year | 12.4±57.8 | 7.2±43.3 | 6.4±34.3 | 8.3±46.5 | 11.2±55.0 | 7.9±45.9 | 8.8±47.8 | 7.2±34.7 | 10.4±52.9 | 9.0±44.5 | 9.1±49.8 | 7.8±40.2 |
| Opium use, nokhod-year^4^ | 3.5±11.5 | 2.5±9.0 | 2.3±8.2 | 2.6±8.7 | 3.1±10.7 | 2.8±9.4 | 2.8±9.5 | 2.6±8.9 | 3.0±10.5 | 2.9±9.5 | 2.8±9.2 | 2.9±9.6 |
| ^1^ Intake density (grams per 1000 kcal)  ^2^ All covariates were associated with nut consumption with p<0.05  ^3^ grams per 1000 kcal/day  ^4^ a local unit for opium consumption that weighs about 200 mg | | | | | | | | | | | | |
